# Canine parvovirus: a predicting canine model for sepsis

**DOI:** 10.1186/s12917-020-02417-0

**Published:** 2020-06-15

**Authors:** F. Alves, S. Prata, T. Nunes, J. Gomes, S. Aguiar, F. Aires da Silva, L. Tavares, V. Almeida, S. Gil

**Affiliations:** 1 Faculty of Veterinary Medicine, ULisboa, Av. Universidade Técnica, 1300-477 Lisboa, Portugal; 2 Veterinary Teaching Hospital, Faculty of Veterinary Medicine, ULisboa, Av. Universidade Técnica, 1300-477 Lisboa, Portugal; 3 CIISA - Centre for Interdisciplinary Research in Animal Health, Faculty of Veterinary Medicine, University of Lisbon, Av. Universidade Técnica, 1300-477 Lisboa, Portugal

**Keywords:** Canine parvovirus, Sepsis, SIRS, PIRO

## Abstract

**Background:**

Sepsis is a severe condition associated with high prevalence and mortality rates. Parvovirus enteritis is a predisposing factor for sepsis, as it promotes intestinal bacterial translocation and severe immunosuppression. This makes dogs infected by parvovirus a suitable study population as far as sepsis is concerned. The main objective of the present study was to evaluate the differences between two sets of SIRS (Systemic Inflammatory Response Syndrome) criteria in outcome prediction: SIRS 1991 and SIRS 2001. The possibility of stratifying and classifying septic dogs was assessed using a proposed animal adapted PIRO (Predisposition, Infection, Response and Organ dysfunction) scoring system.

**Results:**

The 72 dogs enrolled in this study were scored for each of the PIRO elements, except for Infection, as all were considered to have the same infection score, and subjected to two sets of SIRS criteria, in order to measure their correlation with the outcome.

Concerning SIRS criteria, it was found that the proposed alterations on SIRS 2001 (capillary refill time or mucous membrane colour alteration) were significantly associated with the outcome (OR = 4.09, *p* < 0.05), contrasting with the 1991 SIRS criteria (*p* = 0.352) that did not correlate with the outcome. No significant statistical association was found between Predisposition (*p* = 1), Response (*p* = 0.1135), Organ dysfunction (p = 0.1135), total PIRO score (*p* = 0.093) and outcome. To explore the possibility of using the SIRS criteria as a fast decision-making tool, a Fast-and-Frugal tree (FFT) was created with a sensitivity of 92% and a specificity of 29%.

**Conclusion:**

These results suggest that increasing the SIRS criteria specificity may improve their prognostic value and their clinical usefulness. In order to improve the proposed PIRO scoring system outcome prediction ability, more specific criteria should be added, mainly inflammatory and organ dysfunction biomarkers.

## Background

According to the most recent scientific consensus, the term sepsis should be used to describe the organ dysfunction triggered by a deleterious inflammatory host response to infection [1]. After the release of bacteria into the bloodstream as a result of an infection, sepsis only takes place if the host immune system is overpowered resulting in clinically significant bacteraemia [2]. With the goal to establish the correlation between the systemic inflammatory response and sepsis a conference was held in 1991 in which the criteria to assess if a systemic inflammatory response syndrome (SIRS) is taking place were defined [3]. Since the first adaptation of these criteria to animals [4] they have been subjected to a series of modifications and the cut off values slightly vary among investigations. To increase the sensitivity (Se) and specificity (Sp) of these parameters, it is important to use them in association with the clinical judgement when screening animals for sepsis, as they are not sufficiently accurate to establish a definitive diagnosis [5]. For a dog to be diagnosed with SIRS at least two of the following four criteria need to be met: body temperature < 37.8 °C or > 39.4 °C, HR > 140 bpm, RR > 30 breaths/min or PCO2 < 32 mmHg (venous or arterial), WBC < 6000 or > 16,000 cells/μL, or > 3% band neutrophils. Cats need to meet three of the four criteria for SIRS to be diagnosed: body temperature < 37.8 °C or > 39.7 °C, HR < 140 or > 225 bpm, RR > 40 breaths/min, WBC > 19,500 or < 5000 cells/μL, or > 5% band neutrophils [2]. When the circulatory and cellular/metabolic abnormalities are severe enough to increase mortality, septic shock should be considered [1]. This translates into a sepsis associated hypotension non-responsive to intravascular volume expansion, that is, dogs with systolic blood pressure < 90 mmHg or mean arterial pressure < 70 mmHg that only respond to vasopressor therapy [2]. The definition of multiple organ dysfunction syndrome (MODS) has remained unchanged since the first sepsis consensus conference in 1991 [3], where it was defined as *“the presence of altered organ function in an acutely ill patient such that homeostasis cannot be maintained without intervention”*. During sepsis MODS can be considered when at least two organ systems, distant from the infection site, become dysfunctional [2].

In the suspicion of sepsis, vital parameters including temperature (Temp.), heart rate (HR) and respiratory rate (RR) should be measured and compared with the SIRS criteria, as sepsis is diagnosed when the SIRS criteria are fulfilled and an infection is confirmed. The clinical signs tend to be unspecific as they correlate not only with the organ system originally affected by the infectious agent but also with secondary organ dysfunctions. Some dogs may already be in septic shock upon hospital admission. In the initial phase of shock patients´ present with pale mucous membranes, prolonged capillary refill time (CRT) and weak pulses. Later, in the hyperdynamic phase of shock, vasodilation subsists with resulting hyperaemic mucous membranes, a decreased CRT (< 1 s), and strong or bounding pulses. Blood pressure should always be part of physical examination of a suspected SIRS patient as hypotension may be present [5, 6].

The diagnostic approach to a septic animal should include a complete blood count, biochemistry profile and coagulation tests. The hemogram may reveal abnormalities in different cellular lineages. The hematocrit most frequently reveals anaemia secondary to blood loss, haemolysis, oxidative damage and reduced erythrocyte production. Polycythaemia can also be present in hypovolemic animals due to hemoconcentration and splenic contraction. Most animals present leucocytosis and band neutrophils and the blood smear reveal toxic changes to the neutrophils. Due to the immunosuppression and lymphocyte apoptosis, it is also possible for lymphopenia and leukopenia to persist. Platelet consumption and disseminated intravascular coagulation (DIC) resulting in thrombocytopenia is a usual finding. Biochemical abnormalities vary and reflect the organ dysfunctions taking place, either primarily affected by the infection or secondary to the inflammatory state. Common findings include hypoalbuminemia, glycaemia alterations, hypocalcaemia and hyperbilirubinemia [2, 6, 7].

Multiple factors contribute to the development of sepsis in canine parvovirus infections. Cellular destruction, intestinal hypomotility, dysbiosis, gut inflammation and tissue necrosis all contribute to cause disruption of the gastrointestinal mucosal barrier, allowing Gram-negative and anaerobic bacteria translocation from the intestinal lumen to the bloodstream developing bacteraemia [8, 9]. Along with the mucosal barrier disruption, impaired immunity develops increasing the susceptibility to secondary infections. Marked leukopenia (mostly neutropenia and lymphopenia) is often observed in CPV infected dogs, as the virus also targets the mitotically active precursors of leukocytes and lymphoid cells of the bone marrow and lymphoid tissue. Neutropenia and bacteria overload impair the elimination of luminal bacteria from the bloodstream in contrast to healthy animals [8, 10, 11]. With the SIRS progression and the release of inflammatory mediators, the gastrointestinal barrier is compromised again, contributing for the cycle of bacterial translocation [9].

In order to stage septic patients by their risk of mortality/adverse outcome and their potential to respond to treatment, a new stratification system, acronym PIRO, was introduced in 2001. This system allows to stratify patients based on their predisposing conditions, the nature and characteristics of the insult/infection, the extent of the host immune response to it, and the associated organ dysfunction [12]. The purpose of PIRO is to help in the enrolment of individuals in clinical studies and prognosis of septic patients, allowing adapting the therapy offered and improving survival.

The main objective of the current study was to assess the prognostic value of the presenting vital signs as well as to evaluate the possibility of stratifying and classifying septic animals according to a proposed PIRO classification system, using parvovirus infection as a natural model for sepsis study [10]. In addition developing a Fast-and-Frugal tree to reinforce and speed the decision-making process. This methodology could help to assess prognosis and be part of the clinical decision making, as well as helping in the enrolment of study populations in future sepsis studies. For that purpose, parvovirus naturally infected dogs hospitalized in the Infectious Disease Isolation Unit (IDIU) of the Veterinary Teaching Hospital (VTH) of the Faculty of Veterinary Medicine (FMV) of the University of Lisbon (ULisboa), were subjected to two sets of SIRS criteria. The first set, named SIRS 1991, considered SIRS criteria as they were originally proposed [2]. The second set, named SIRS 2001, keeps the same criteria of SIRS 1991 plus capillary refill time or mucous membrane colour alteration [12] to attempt to improve the criteria specificity. Then patients were classified by a PIRO classification system adapted from humans to dogs. Finally, all individual variables of PIRO, the total PIRO score and both SIRS criteria were correlated with the outcome.

## Results

### Sample characterization

All dogs included in this study were evaluated by a veterinarian from the VTH.

The target population included dogs hospitalized in the IDIU from November 2013 until June 2019, with a positive laboratory diagnosis of canine parvovirosis either by ELISA or PCR faecal antigen detection, with clinical exam, haemogram, biochemistry records and known outcome, discharge or death.

All dogs that did not fulfil the previous inclusion criteria or had concomitant diseases able to induce gastrointestinal signs were excluded.

The sample size was 72 dogs. Concerning the outcome 59 (81.9%) dogs were discharged and 13 (18.1%) dogs died. Regarding gender 42 (58.3%) were male and 30 (41.7%) female. The majority of the dogs, 52 (72.2%), fell under the described susceptible age group of over 6 weeks and under 6 months, 12 (16.7%) dogs were over 6 months old, 5 (6.9%) were under 6 weeks old and 3 (4.2%) were of unknown age. As far as vaccination status is concerned, most dogs, 37 (51.3%) had no vaccination history, 29 (40.3%) an incomplete vaccination programme, 4 (5.6%) an unknown vaccination history and only 2 (2.8%) were considered to have a complete vaccination status for parvovirus infection. Most dogs, 31(43.1%) had no defined breed.

### SIRS criteria

Table 1 gathers all leucocyte counts, a selection of clinical examination parameters (Temperature, Heart Rate and Respiratory Rate), all individual variables of PIRO (P=Predisposition, I=Infection, R = Response, O=Organ Dysfunction), the total PIRO score and both SIRS criteria for survivors and non-survivors dogs. No significant statistical association was found between the fulfilment of the SIRS 1991 criteria and the outcome (*p* = 0.352). However, when considering the new criteria SIRS 2001 requiring CRT or mucous membrane colour alteration, a statistical association was found between SIRS 2001 criteria and the outcome (OR = 4.09, *p* = 0.0242). This suggests that dogs fulfilling the SIRS 2001 criteria upon admission were approximately 4 times more likely to die than those who did not (Table 2).

**Table 1:** Clinical and laboratory parameters, SIRS 1991, SIRS 2001 and total PIRO score for survivors and non-survivors

Outcome	Animals (***n***)	HR ($\overline{\boldsymbol{x}}$ ±SD)	RR ($\overline{\boldsymbol{x}}$ ±SD)	T °C ($\overline{\boldsymbol{x}}$ ±SD)	Leucocytes (cells/μl) ($\overline{\boldsymbol{x}}$ ±SD)	SIRS 1991 (***n***)	SIRS 2001 (***n***)	P ($\overline{\boldsymbol{x}}$ ±SD)	I	R ($\overline{\boldsymbol{x}}$ ±SD)	O ($\overline{\boldsymbol{x}}$ ±SD)	Total PIRO score ($\overline{\boldsymbol{x}}$ ±SD)
Survivors	58	146.88 ± 36.32	35.83 ± 13.07	38.65 ± 0.87	9.69 ± 7.77	36	14	7.32 ± 1.09	1	3.93 ± 2.66	0.18 ± 0.39	12.43 ± 2.90
Non-survivors	14	146.81 ± 35.12	35.58 ± 14.49	38.56 ± 0.89	10.71 ± 8.22	11	8	7.21 ± 1.17	1	3.77 ± 2.65	0.21 ± 0.41	12.19 ± 2.90

Legend: HR - Heart Rate (beats per minute); RR - Respiratory Rate (breaths per minute); T - Temperature (degrees Celsius);

Leucocytes (cells per microliter); SIRS 1991 Criteria; SIRS 2001 Criteria; P - Predisposition; I - Infection; R - Response;

O - Organ dysfunction; Total PIRO score (Total PIRO scoring system)

**Table 2:** Fisher’s exact test results of the correlation between the different SIRS criteria and the outcome

	*p*-value	Odds Ratio	95% confidence interval
SIRS 1991	0.352	2.21	0.507–13.735
SIRS 2001	0.0242	4.09	1.044–17.109

Significance limits: *p*-value ≤0.05

Legend: SIRS 1991 Criteria; SIRS 2001 Criteria

### Predisposition

For the current study, the predisposing factors included were age, breed and vaccination status (Table 3). The sample was composed mainly by undefined breed dogs (43.1%) and 14 dogs were considered to have a breed predisposition for Parvovirus enteritis (10 Labrador, 2 German Shepherd, 1 Rottweiler and 1 Alaskan Malamute) [17]. Most dogs (72.2%) were aged between 6 weeks and 6 months and the majority of the animals included had no vaccination history (51.3%) or an incomplete/incorrect vaccination (40.3%). As far as age and vaccination status are concerned, this sample reflects descriptions in the literature about parvovirus infection predisposition, with young unvaccinated dogs being the most susceptible to infection. In the present study no significant statistical association was found between predisposition and outcome (*p* = 1) (Table 4).

**Table 3:** Proposed predisposition (P) scoring criteria for parvovirus infected dogs, considering age, breed and vaccination status [11, 13–16]

	Parameters	Score
**Age**	< 6 weeks	**1**
	> 6 weeks ≤6 months	**3**
	> 6 months	**2**
**Breed**	Toy Poodle and Cocker Spaniel	**1**
	Undefined	**2**
	Rottweiller, Labrador Retriever, American Staffordshire,Terrier, German Sheperd, Alaskan Malamute	**3**
**Vaccination Status**	Complete Primary Vaccination	**0**
	Incomplete/Incorrect Primary Vaccination	**2**
	Not Vaccinated or Unknown Vaccination Status	**3**

**Table 4:** Fisher’s exact test results of the correlation between the different PIRO variables and the outcome

	p-value	Odds Ratio	95% confidence interval
Predisposition ^a^	1	0.67	0.013–6.329
Response ^b^	0.1135	0.294	0.065–1.409
Organ Dysfunction ^c^	0.1135	0.294	0.065–1.409
Total PIRO ^d^	0.093	0.161	0.004–1.226

Significance limits: p-value ≤0.05

Legend: ^a^ Lower-half score ranging from 0 to 5: Non-survivors (*N* = 1); Survivors (*N* = 6); Upper-half score ranging from 6 to 10: Non-survivors (*N* = 13); Survivors (*N* = 52)

^b^ Lower-half score ranging from 0 to 6: Non-survivors (*N* = 9); Survivors (*N* = 50); Upper-half score ranging from 7 to 12: Non-survivors (N = 5); Survivors (*N* = 8)

^c^ Lower-half = 0: Non-survivors (N = 9); Survivors (N = 50); Upper-half score = 1: Non-survivors (N = 5); Survivors (N = 8)

^d^ Lower-half score ranging from 0 to 10: Non-survivors (N = 1); Survivors (*N* = 19); Upper-half score ranging from 11 to 20: Non-survivors (*N* = 13); Survivors (*N* = 39)

### Infection

In our study parvovirus was considered to be the only infection inducing element and, since all the animals were considered to have the same infection score of “1”, its’ correlation with the outcome was not statistically evaluated.

### Response

**Fig. 1:**
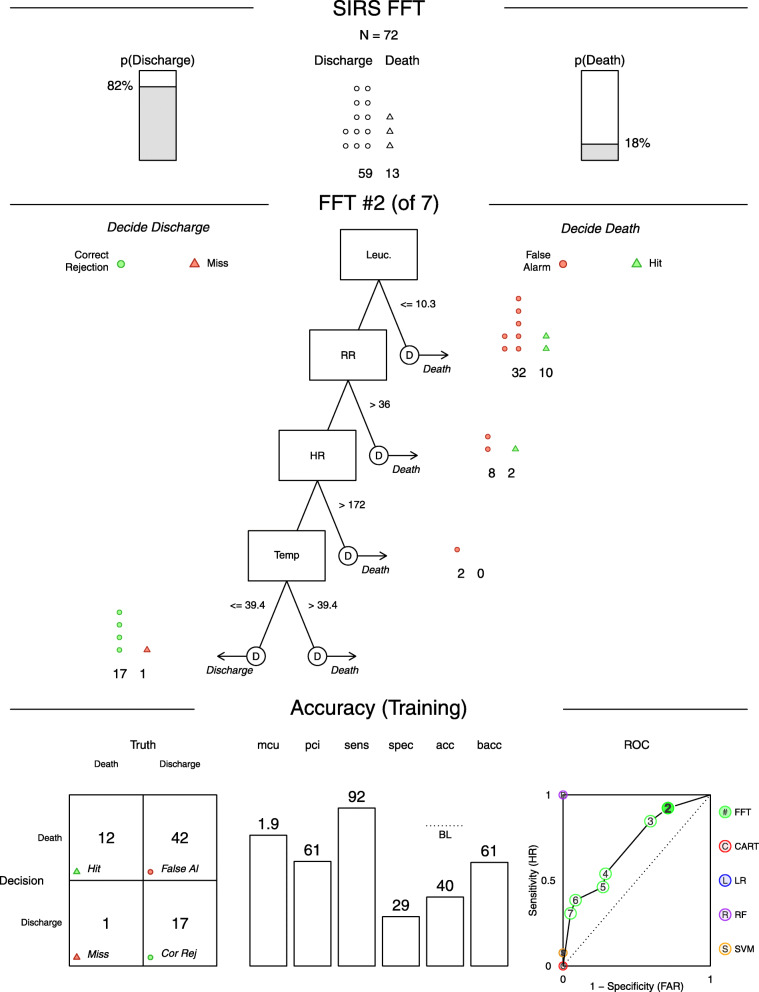
.

To explore the possibility of using the SIRS criteria as a fast decision-making tool, a Fast-and-Frugal tree (FFT) (Fig. 1) was created using the criteria considered to characterize the Response (R) element. This FFT revealed a sensitivity of 92% and a specificity of 29%.

Factors considered for the characterization of the host inflammatory response were the SIRS diagnosis criteria (HR, RR, T and leucocytes count) (Table 5). No significant statistical association was found between response (R) and outcome (*p* = 0.1135) (Table 4).

**Table 5:** Proposed response (R) scoring criteria for parvovirus infected dogs [2, 14, 18]

Criteria	Score			
	0	1	2	3
**T (°C)**	37.8–39.4	39.5–40.4	36–37.7 or 40.5–41.4	< 36 or > 41.4
**HR (bpm)**	60–140	141–150	151–170	< 60 or > 171
**RR (bpm)**	10–30	31–40	41–50	> 50 ou < 10
**Leucocytes Count (cells/μL)**	6000–16.000	4.200–5.999 or 16.001–20.800	2.940–4.199 or 20.801–27.040	< 2.939 or > 27.041

Legend: T - (Temperature degrees Celsius); HR - Heart Rate (beats per minute); RR - Respiratory Rate (breaths per minute); Leucocytes (cells per microliter)

### Organ dysfunction

Regarding Organ Dysfunction from the 72 dogs enrolled in this study, only 13 were considered to have some kind of organ dysfunction, each scoring “1” (Table 6). Hepatic dysfunction was the most frequent cause of organ dysfunction with 8 dogs falling under this classification, 6 due to hypoalbuminemia and 2 with elevated ALP. Regarding these 8 dogs 4 were deceased and the other 4 survived. From the remaining dogs two were considered to have renal dysfunction, one with a creatinine increment and the other with both creatinine and urea elevation. Only the first of these dogs died. Three dogs with low platelet count were included on the coagulation dysfunction group and they all survived. None of the animals included showed signs of cardiovascular or respiratory dysfunction.

**Table 6:** Proposed organ dysfunction (O) scoring criteria. Each evidence of organ dysfunction equals to 1 point

Dysfunction	Criteria
**Renal**	Creatinine> 1.64 mg/dl; Creatinine> 1.64 mg/dl and Urea> 56 mg/dl
**Cardiovascular**	Hypotension requiring vasopressor drugs administration
**Respiratory**	Need for oxygen or ventilation supply, ARDS
**Hepatic**	Bile acid> 25 μmol/L (post-prandial) and/or Bilirubin> 0,41; ALT> 130 U/L 37 °C e ALP > 337 U/L 37 °C; Albumin < 2.1 g/dl
**Coagulation alterations**	Platelets≤100.000/μL

Legend: mg/dl (milligram per decilitre); g/dl (gram per decilitre); ARDS (Acute Respiratory Distress Syndrome); ALT (alanine aminotransferase); ALP (alkaline phosphatase); U/L 37 °C (units per litre at 37 degrees Celsius); μL (microliter)

The considered values correspond to the upper limit considered by VTH Clinical Analysis Laboratory (where all the blood samples were analysed)

## Discussion

Since it was first adapted for veterinary medicine, SIRS has been widely used by clinicians and researchers to diagnose sepsis in animals. On a 2006 sepsis survey, 80% of veterinarians acknowledged to use SIRS criteria [19]. A pioneer study to propose SIRS classification criteria for animals and to assess their ability to diagnose sepsis accurately was carried out by Hauptman et al. [5]. The meeting of at least two of the four criteria method revealed a Se of 97% and Sp of 64%. Even though the high sensitivity found indicates that almost all septic animals could be detected, the low specificity implies an over diagnosing of sepsis due to 36% false positives (FP).

In an attempt to enhance the criteria specificity in sepsis diagnosis, the 2001 SCCM/ESICM/ACCP/ATS/SIS International Sepsis Definitions Conference Task Force proposed a list of possible signs of inflammatory response to infection that could be added to the existing SIRS criteria, hence augmenting their specificity [12]. In the present study one of the proposed variables was added to the existing SIRS criteria in order to create a new set of criteria denominated SIRS 2001. Accordingly, the SIRS criteria would only be applied upon an increased capillary refill time or a mucous membrane colour alteration.

In our study, when the new classification criteria were applied, a significant statistical association with the outcome was found (OR = 4.09, *p* < 0.05). In fact, according to our results, dogs that met the SIRS 2001 criteria on admission were about 4 times more likely to die than those who did not (Table 2). This improved mortality forecast in medical emergency ward canine patients may be due to an increased specificity of the criteria applied, as they might be indicators of hemodynamic instability and tissue perfusion compromise. Even though none of the proposed parameters are specific for sepsis, they can be indicators of an onset of organ dysfunction, which is consistent with the most recent sepsis definition, as a “life-threatening organ dysfunction caused by a dysregulated host response to infection”, thus helping to increase the specificity of the diagnosing criteria [1].

Sepsis predisposition takes into account all the factors that are present before the onset of sepsis and that may influence the outcome upon an infectious insult. Apart from genetic factors, both age and medical co-morbidities were reported in various studies to be associated with hospital mortality [20–22]. Further studies are needed to increase knowledge about the influence of predisposing factors in the systemic inflammatory response of animal species. One study concluded that geriatric dogs had a weaker IL-10 production upon bacterial LPS stimulation, which may turn into an exacerbated inflammatory response and greater risk of mortality when compared to younger dogs [23]. Breed has also been shown to influence the inflammatory response. Nemzek et al. [17] found that CPV highly susceptible breeds such as Rottweiler and Doberman pinscher showed an increase in Tumour Necrosis Factor-α (TNF-α) production in response to LPS stimulation when compared to mixed breeds.

In the current study, the predisposing factors investigated were age, breed and vaccination status (Table 3). The sample was composed mainly by undefined breed dogs (43.1%) and 14 dogs were considered to have a breed predisposition for CPV enteritis (10 Labrador, 2 German Shepherd, 1 Rottweiler and 1 Alaskan Malamute) [17]. Most dogs (72.2%) were aged between 6 weeks and 6 months and most of the animals had no vaccination history (51.3%) or an incomplete/incorrect vaccination (40.3%). As far as age and vaccination status are concerned, this sample reflects what has been previously reported about CPV epidemiology, with young unvaccinated dogs being the most susceptible to infection [8].

No significant statistical association was found between predisposition and outcome (*p* = 1) (Table 4). This result differs from most human medicine studies that have shown a significant statistical association between predisposition and outcome, with Granja et al. [20] and Howell et al. [21] reporting a strong correlation with mortality (*p* < 0.001). When considering the predisposition (P) discriminatory ability for predicting outcome, a poor to fair accuracy was described by Rathour et al. [22] reporting an area under the receiver operating characteristics curve (AUC-ROC) of 0.79, while Granja et al. [20] reported an AUC-ROC of just 0.66. These results suggest that predisposition alone is not a good outcome prediction element but, since it is associated with the outcome, it should be included in the total PIRO classification in order to improve its overall accuracy. Population characteristics including an overrepresentation of animals within the susceptible age group and underrepresentation of death among the susceptible breeds may also have contributed for the discrepancy between the results obtained in this study and the ones previously cited.

On the other hand, our findings come into agreement with the results observed by Kalli et al. [24] on CPV infected dogs, where no correlation between any particular breed or age group and the outcome was found. Only purebreds were 2.5 times more likely to develop the disease. It might be the case that the parameters chosen to characterize the dogs´ predisposition in this study, for example breed, were not the most suitable to evaluate the predisposition influence on sepsis mortality.

For the current study CPV was considered to be the only infection inducing element and, since all the dogs were considered to have the same infection score of “1”, its’ correlation with the outcome could not be statistically evaluated. Moreover, this was a limitation of the study, because whenever a dog exhibited clinical examination results, haematological and serum biochemistry compatible with parvovirosis, other agents of acute gastroenteritis of infectious or parasitic origin were not investigated. Yet when the pattern of clinical signs or haematological and serum biochemistry suggested the possibility of another disease, these dogs were always tested and rejected according to the exclusion criteria described in Methods.

One interesting use of SIRS criteria is the possibility to construct a Fast-and-Frugal Tree (FFT) to predict sepsis outcome, thus helping to speed decision-making. Figure 1 represents one FFT that may be helpful to characterize and score the response (R) element of the PIRO system. This FFT was not tested on another sample and so the results may only reflect the tree’s performance for this particular sample [25]. This FFT has a sensitivity of 92% and a specificity of 29%. Sensitivity was primed over specificity in the making of this FFT, as it is more important to have a higher sensitivity when considering life-threatening conditions like sepsis, in order to reduce the number of false negatives and the risk of missing critically ill animals. The high sensitivity obtained means a good prediction might be expected 92% of the times, when attributing a good prognosis. The low specificity observed implies that 71% of the dogs would be wrongly given a poor prognosis but would end up surviving. Using this FFT for prognosis attribution may imply a high number of false alarms. However, while the consequences of a low specificity would be a closer monitoring of not so critically ill animals, if specificity was privileged over sensitivity, we would risk attributing a good prognosis to critically ill dogs. This could lead to a less rigorous clinical monitoring of these animals, reducing their survival chances. Decision aids, like this FFT, may contribute to define clinical parameters and to establish cut off values for them, making them a valuable tool in sepsis research.

The inflammatory response is also accountable for the clinical changes observed during sepsis and can have predictive value. Variables proven to be related with mortality include heart and respiratory rate, leucocyte and band neutrophils count [10, 19–21]. In this study the factors used for the characterization of the host inflammatory response were the SIRS diagnosis criteria: HR, RR, T and leucocytes count (Table 5). No significant statistical association was found between response (R) and outcome (*p* = 0.1135) (Table 4). These results differ from what has been reported in human medicine studies, in which response was positively associated with mortality. Granja et al. [20] and Howell et al. [21] both observed a strong correlation between response and outcome, with a *p* = 0.002 and a *p* < 0.001 being reported respectively. On another study the only two response variables related with hospital mortality were increased respiratory rate (> 20 breaths/min) and bandemia (> 5% immature band neutrophils). In that study a fair outcome prediction accuracy was reported for the response element, with an AUC-ROC = 0.74 [22].

The same limitations described above for the SIRS criteria may be pointed (higher respiratory and heart rate in puppies and lower leukocyte count due to viral destruction) as the same criteria were used to characterize both [26, 27]. To improve the outcome prediction ability of the response element, biochemical markers of inflammation such as inflammatory cytokines (IL-6 and TNF), C-Reactive Protein (CRP) or coagulation proteins should be included in future investigations [21, 22]. The retrospective nature of the present study made it impossible to include biochemical markers of inflammation, as they are not part of routine biochemical analyses.

The presence of organ systems dysfunction caused by a deleterious inflammatory response is what differentiates sepsis from an infection, and the presence of organ failure has been shown to correlate with the outcome. Yet in this study, no statistical association was found between organ dysfunction and outcome (*p* = 0.1135) (Table 4), diverging from the results reported in other studies.

Kenney et al. [28] reported that cardiovascular dysfunction, coagulation dysfunction, renal dysfunction (*p* < 0.001) and respiratory dysfunction (*p* < 0.01) in dogs, were all independently associated with the outcome. They reported that mortality rate rose as the number of organ systems affected increased. For the overall organ dysfunction score and its’ correlation with the outcome, Rathour et al. [22] described a good outcome prediction accuracy, with an AUC-ROC = 0.81. A statistical association between overall organ dysfunction score and outcome was also reported by Granja et al. [20].

From the 72 dogs enrolled in this study, only 13 were considered to have some kind of organ dysfunction, each scoring “1” (Table 1). Hepatic dysfunction was the most frequent cause of organ dysfunction with 8 dogs falling under this classification, 6 due to hypoalbuminemia and 2 with elevated ALP. Of these eight dogs, four died and the other four survived. Hypoalbuminemia is a rather nonspecific parameter to access hepatic function especially during sepsis, as increased vascular permeability and shifting to acute phase proteins production also contribute to the albumin decrease [6, 13]. Considering that our study population was composed of CPV infected dogs, hypoalbuminemia may even be less specific as an hepatic function marker, with the most probable cause for hypoalbuminemia being gastrointestinal protein loss. Alkaline phosphatase (ALP) is not directly related with impaired liver function, even though it can be increased during sepsis due to cholestasis [13]. The ALP increment was slight in both dogs suggesting it could result from individual variation. The low specificity of the variables chosen to evaluate liver function and the reported inconsistency of hepatic dysfunction as a mortality predictor, may have affected the results of the present study.

Two remaining dogs were considered to have renal dysfunction, one with a creatinine increment and the other with both creatinine and urea raise. Only the first of these dogs died. Based on renal dysfunction consensus the criteria that should be included in the definition of renal dysfunction include serum creatinine concentration, glomerular filtration rate and urine output. In the present study only creatinine and urea were considered as renal dysfunction markers, which may have impaired the ability to identify the presence of renal dysfunction on this sample [29]. Nonetheless, Kenney et al. [28] reported a strong association between renal dysfunction and outcome even when using serum creatinine concentration as the only renal dysfunction marker.

Finally, three dogs with low platelet count were included on the coagulation dysfunction group and they all survived. Even though coagulation dysfunction has been independently associated with mortality, there are still conflicting results when it comes to platelets count. While Hauptman et al. [5] reported thrombocytopenia as a good sepsis marker, Laforcade et al. [30] found no significant difference on platelet counts between dogs with sepsis and the control group. The measurement of other coagulation markers like prothrombin time (PT), partial thromboplastin time (PTT), fibrin degradation products (FDP) and D-dimer (DD) concentrations, could have helped on the detection of more animals suffering from coagulation disorders.

None of the animals investigated showed signs of cardiovascular or respiratory dysfunction so it was impossible to assess their influence on the outcome. The fact that the criteria proposed to characterize both of these organ systems dysfunctions were rather subjective, as they require clinical judgment, may contribute for the results obtained. Future studies should consider other cardiovascular and respiratory function markers. As far as the respiratory function is concerned there are, since 2007, veterinary medicine criteria to assess the presence of acute lung injury and acute respiratory distress syndrome [31]. Helpful clinical exams for the characterization of cardiac dysfunction should include echocardiography as it would identify the presence of biventricular dilatation or a decreased ejection fraction [32]. Other classification systems like the Sequential Organ Failure Assessment (SOFA) may be used to improve the scoring of organ dysfunction (O) and contribute to a more accurate overall PIRO scoring system.

In the present study no significant statistical association was found between the total PIRO score and the outcome (*p* = 0.093). Different studies described a fair to good outcome prediction capacity. Rubulotta et al. [33], reported an area under the curve of 0.696. Even though this result defines only a fair mortality prediction ability for PIRO, it was equivalent to the AUC published for other scoring systems at the time, which ranged from 0.6 to 0.7 [34].

Howell et al. [21] conducted a study that validated PIRO’s utility. The proposed classification system was created based on the variables found to be independently statistically significant associated with mortality, which increased the outcome prediction accuracy. The scoring system was applied to a sample group and to two validation cohorts. Results revealed that mortality was strongly related to an increased PIRO score in all groups, with an AUC-ROC of 0.9, 0.86 and 0.83, respectively. On another study carried out by Nguyen et al. [35] PIRO performed better (AUC-ROC = 0.71) than MEDS (Mortality in Emergency Department Sepsis) and was comparable to the APACHE II (Acute Physiology and Chronic Health Evaluation). Li [36] conducted a study on community-acquired sepsis in which an AUC of 0.90 for PIRO 28-day mortality prediction was reported, outperforming the APACHE II. Recently, results reported by Songsangjinda and Khwannimit [37] on septic patients admitted over a 9 year period, showed that the Moreno PIRO had the best discriminating capacity with an AUC-ROC of 0.835, outperforming all the other classification systems and only closely followed by SOFA (AUC-ROC = 0.828). The best discriminative capacity for PIRO mortality prediction was described by Rathour et al. [22] with an AUC of 0.94.

The mortality rate on this study was 18.1%, which may indicate an underrepresentation of the death group thus contributing to the results obtained, as sepsis mortality rates have been reported to go up to 68% and reaching 90% in the presence of septic shock [28, 38]. The relatively narrow range of the total PIRO score may also have contributed to a worse outcome prediction capacity. Future studies should include more parameters to better characterize the systemic inflammatory response and distinguish animals based on severity of illness. The individual assessment of the parameters and how they independently affect the outcome, as well as the addition of other biomarkers [39, 40], that may help to characterize the inflammatory response, could contribute to a better outcome prediction accuracy of the proposed PIRO scoring system.

Even though no significant statistical association was found between the total PIRO score and the outcome in this study, this is the first version of the Dog PIRO Model and may serve as reference for future studies.

## Conclusions

The present study stands as a contribution for the development of a robust and validated classification system for sepsis in dogs. To our knowledge, this is the first study to propose and assess the implementation of a PIRO classification system in dogs, adapting it from what has been used in human medicine and testing it on a sepsis predisposed population of parvovirus naturally infected dogs.

Concerning the SIRS criteria, this study confirms the potential of including animals with altered mucous membrane colour or prolonged capillary refill time. This increased the system specificity, allowing for the identification of a statistical association with the outcome. This was an attempt to address the need for more specific sepsis related systemic inflammatory criteria. To explore the possibility of using the SIRS criteria as a fast decision-making tool, a Fast-and-Frugal tree (FFT) was created.

In our study no significant statistical association was found between PIRO’s elements and outcome. Nevertheless, it demonstrates that defining and applying a classification system for sepsis suspected canine patients is possible. Future studies should include more inflammation and organ dysfunction biomarkers, that may help to characterize each of the PIRO’s components and consequently its´ overall performance. Further adjustments to overcome the weakness reported in this study are necessary to improve its´ outcome prediction capacity and to turn it into a useful tool in the clinical management of sepsis.

## Methods

### Exclusion criteria

All patients also diagnosed with other viral infections compatible with acute gastroenteritis, namely, distemper (*N* = 1), infectious canine hepatitis (*N* = 2) and canine coronavirus (*N* = 3), were excluded from the study, amounting to six dogs with dual viral infectious diseases. The same criteria applied to all dogs with concomitant internal parasitic infections (*N* = 9). The remaining 24 dogs excluded from the study population (*N* = 102) missed either the hemogram, biochemistry results, some clinical examination parameters or the outcome.

### Classification criteria

In order to evaluate if a SIRS was taking place, the animals were subjected to two sets of criteria. The first set was denominated SIRS 1991 based upon the originally proposed SIRS criteria, still currently used in clinical practice. The values considered for each variable are those described above in the background. The second set of criteria, designated SIRS 2001, is a combination of SIRS 1991 plus CRT or mucous membrane colour alteration. SIRS 2001 was created in an attempt to increase the specificity of said criteria, as suggested by Levy et al. [12].

The proposed criteria applied for the PIRO scoring, and for each of its’ individual components - P, R and O - were extrapolated from an array of bibliographic references, from human and veterinary medicine studies. These criteria were already compiled in a previous study [14]. The parameters proposed for each of the PIRO’s components are described in Tables 3, 5 and 6. For predisposition (P) age, breed and vaccination status were considered. Response (R) was characterized by temperature, heart rate, respiratory rate and leucocytes count. Organ dysfunction (O) by biochemical and clinical markers of renal, cardiovascular, respiratory, hepatic and coagulation system dysfunction. Since dogs naturally infected with canine parvovirus were enrolled in this study, all animals had the same infection (I) score (equal to 1). The total PIRO score was obtained by adding all PIRO’s components score.

All dogs that participated in this study were client-owned animals and joined the study after owner’s written consent and Ethical Committee approval (CEBEA).

### Statistical analysis

To evaluate the correlation between the fulfilment of the SIRS criteria and the outcome, all dogs were classified according to the original SIRS 1991 and to the proposed SIRS 2001 criteria. Fisher’s exact test was used. A 95% confidence interval was considered.

To assess the correlation between the PIRO scoring and the outcome, the scores for total PIRO and each of its’ components were split into two groups, a Fisher’s exact test requirement. For each variable, one of the groups contained the dogs in the lower half of the values and the other comprised the animals in the upper half. A 95% confidence interval was used in Fisher’s exact test.

## Data Availability

The datasets used and/or analysed during the current study are available from the corresponding author on reasonable request.

## Abbreviations

ALP: Alkaline Phosphatase
ALT: Alanine Transaminase
APACHE: Acute Physiology and Chronic Health Evaluation
ARDS: Acute Respiratory Distress Syndrome
AUC-ROC: Area Under the Receiver Operating Characteristics Curve
bpm: beats per minute
°C: degrees Celsius
CEBEA: Committee for Ethics and Animal Welfare
CPV: Canine Parvovirus
CRP: C-Reactive Protein
CRT: Capillary Refill Time
DD: D-dimer
FDP: Fibrin degradation Products
FFT: Fast-and-Frugal Tree
FMV: Faculty of Veterinary Medicine
HR: Heart Rate
IDIU: Infectious Disease Isolation Unit
MEDS: Mortality in Emergency Department Sepsis
mmHg: millimeter of mercury
MODS: Multiple Organ Dysfunction Syndrome
OR: Odds Ratio
PCO2: Partial Pressure of Carbon Dioxide
PIRO: Predisposition, Infection, Response, Organ Dysfunction
PT: Prothrombin Time
PTT: Partial Thromboplastin Time
RR: Respiratory Rate
SIRS: Systemic Inflammatory Response Syndrome
Se: Sensibility
Sp: Specificity
SOFA: Sequential/ Sepsis-related Organ Failure Assessment
T: Temperature
TF: Tissue Factor
TNF: Tumor Necrosis Factor
ULisboa: University of Lisbon
VTH: Veterinary Teaching Hospital
WBC: White Blood Cells
μL: microliter
